# Anti-tumor activities and apoptotic mechanism of ribosome-inactivating proteins

**DOI:** 10.1186/s40880-015-0030-x

**Published:** 2015-07-17

**Authors:** Meiqi Zeng, Manyin Zheng, Desheng Lu, Jun Wang, Wenqi Jiang, Ou Sha

**Affiliations:** School of Medicine, Shenzhen University Health Science Center, Shenzhen, 518060 Guangdong People’s Republic of China; School of Medicine, Shenzhen University, Shenzhen, 518060 Guangdong People’s Republic of China; State Key Laboratory of Oncology in South China, Guangzhou, Guangdong 510060 People’s Republic of China; Collaborative Innovation Center of Cancer Medicine, Guangzhou, 510060 Guangdong People’s Republic of China

**Keywords:** Ribosome-inactivating protein (RIP), Anti-tumor, Apoptosis, Cancer

## Abstract

Ribosome-inactivating proteins (RIPs) belong to a family of enzymes that attack eukaryotic ribosomes and potently inhibit cellular protein synthesis. RIPs possess several biomedical properties, including anti-viral and anti-tumor activities. Multiple RIPs are known to inhibit tumor cell proliferation through inducing apoptosis in a variety of cancers, such as breast cancer, leukemia/lymphoma, and hepatoma. This review focuses on the anti-tumor activities of RIPs and their apoptotic effects through three closely related pathways: mitochondrial, death receptor, and endoplasmic reticulum pathways.

## Introduction

Ribosome-inactivating proteins (RIPs) are a family of enzymes that inhibit the eukaryotic ribosome via *N*-glycosidase activity, by which they cleave a specific adenine residue from the 28S RNA within the 60S ribosomal subunit, therefore inhibiting protein synthesis [1, 2]. In addition to their effect on ribosomal RNA (rRNA), some RIPs display a variety of anti-microbial activities in vitro, including anti-fungal, anti-bacterial, and broad-spectrum anti-viral properties against both human and animal pathogens.

Ribosome-inactivating proteins were initially discovered in the castor oil plant *Ricinus communis*, from which ricin was isolated. RIPs are widely distributed among higher plants, and a few have been found in several fungi and bacteria. Plant RIPs are classified into three main categories based on their physical properties. Type I RIPs are single-chain proteins of approximately 30 kDa with *N*-glycosidase activity, including trichosanthin (TCS) and cucurmosin [3]. Type II RIPs, such as ricin and abrin, comprise two different domains: a 30-kDa enzymatic A-chain (similar to type I RIPs) linked to a slightly larger B-chain with lectin properties and specificity for sugars possessing galactose-like structures [3]. Thus far, type III RIPs, also considered atypical type I RIPs, have only been described in maize and barley, and the function of their extra domains remains unknown [4]. Therefore, the division of RIPs into types I and II RIP is now favored.

Over the past decade, RIPs appeared to be a great research interest due to their potential use in cancer therapy. Some RIPs exhibit strong toxicity towards cancer cells and low toxicity towards normal cells; they impede or inhibit tumor growth mostly via apoptosis, but the exact mechanism remains poorly understood. The aim of this study was to summarize the anti-tumor activities of RIPs and their possible apoptotic mechanisms to hopefully provide new insights for cancer research and treatment.

## Anti-tumor activity

### Effects of RIPs on breast cancer

The type I RIPs TCS, momordica anti-human immunodeficiency virus (HIV) protein of 30 kDa (MAP30), gelonium anti-HIV protein of 31 kDa (GAP31), gelonin, marmorin, and α-momorcharin (α-MMC) have been shown to negatively affect the growth of breast tumor cells in vitro and in vivo [5–7]. TCS inhibits cell viability, causes cell cycle arrest, and significantly reduces tumor volume and weight by inducing apoptosis through caspase-8 and caspase-9 in breast tumor cells [5]. MAP30, which shares 59% sequence similarity with TCS, effectively inhibits human breast cancer MDA-MB-231 cells through down-regulating the expression of human epidermal growth factor receptor-2 (HER2), similarly to GAP31 [6]. HER2 overexpression is observed in approximately 30% of all human breast cancers, and HER2-overexpressing tumor cells may be less sensitive to chemotherapy; in this case, the combination of MAP30 and GAP31 could represent a therapeutic strategy [7]. HER2 and fibroblast growth factor-inducible 14-kDa protein (Fn14) are frequently co-expressed in human breast tumors, and HER2 directly induces increase in Fn14 expression, therefore sensitizing tumor cells to an immunotoxin generated by fusing Fn14 antibodies to recombinant gelonin (designated hSGZ) [8]. Indeed, hSGZ can rapidly internalize and deliver recombinant gelonin (rGel) to the cytosol of tumor cells, where it enzymatically blocks protein synthesis. Because Fn14 enhances breast cancer cell migration and invasion, a question of whether there is a way to damage tumor cells while reducing Fn14 expression was raised. We assume that MAP30 and hSGZ used together might achieve a better outcome, and breast tumor cells can be sequentially treated with MAP30 and hSGZ; MAP30 would decrease HER2 expression and lead to reduced Fn14 expression, then hSGZ would target Fn14-positive cells and exert its function without increasing the invasive capacity of tumor cells. However, this hypothesis remains to be verified by appropriate experiments.

Many cell membrane receptors are expressed at low levels in normal cells but are highly expressed in tumor cells. Estrogen receptor α (ERα) is expressed in approximately 75% of breast cancer tissues at higher levels compared with those in normal breast tissues (*P* = 0.001) [9]. In ERα-positive breast cancer cells, the ERα-mediated signaling pathway is involved in the inhibitory action of marmorin on proliferation; many drugs target ERα. Marmorin inhibits angiogenesis by lowering the viability of human umbilical vein endothelial cells in vitro; therefore, it was suggested that marmorin might starve tumors to death by reducing the amount of blood vessels in vivo [10]. Marmorin also induces DNA damage and endoplasmic reticulum stress, resulting in the induction of apoptosis in mice bearing MDA-MB-231 tumor xenografts [10].

Ribosome-inactivating proteins have the potential to become innovative anti-tumor agents, but they also possess toxic adverse effects, including severe systemic anaphylaxis, immunogenicity, and toxicity. To reduce the undesirable effects and achieve better therapeutic efficacy, Deng et al. [11] modified α-MMC with polyethylene glycol (PEG) to explore the anti-tumor efficacy on breast carcinoma; they demonstrated that α-MMC PEGylation extends the half-life of α-MMC and mitigates non-specific toxicity. Indeed, α-MMC-PEG exhibited improved anti-tumor efficacy with tolerable toxic reactions.

### Effects of RIPs on leukemia and lymphoma

Trichosanthin significantly inhibits the proliferation of various leukemia and lymphoma cell lines [12]. Notably, TCS can damage leukemia and lymphoma cells through different mechanisms according to the cell type. TCS induces apoptosis in T-lymphocyte cell lines, but inhibits growth of B-lymphocyte cell lines via S-phase cell cycle arrest [12]. It has been suggested that cucurmosin is more potent than TCS in killing the chronic myelogenous leukemia K562 cells; both cucurmosin and TCS down-regulate P210^Bcr-Ab1^ and inhibit tyrosine kinase, resulting in cell growth suppression [13]. Cucurmosin also inhibits proliferation and induces apoptosis in tumor cells; interestingly, cucurmosin combined with trans-retinoic acid or arsenic trioxide was shown to synergistically increase these effects on the human acute promyelocytic leukemia NB4 cell line [14].

Articulatin-D, the first cytotoxic RIP with a B-chain lacking sugar-binding activity, has been shown to highly inhibit leukemia and lymphoma cells in vitro; the highest toxicity was obtained with Jurkat cells, followed by Molt-4, U-937, HL-60, and Raji cells [15]. With its special physical properties, articulatin-D is a good candidate for the synthesis of immunotoxins capable of efficiently and specifically killing tumor cells.

Immunotoxins are emerging targeted agents composed of a toxin fragment and an antibody/cytokine. Saporin and rGel have been widely used to construct immunotoxins, which have been reported to be useful in cancer treatment by multiple studies [16–18]; several such molecules have been evaluated clinically [19, 20]. It is feasible to locate cancer cells through membrane proteins CD22, CD7, CD19, and CD38, and corresponding antibody HB22.7, HB2, BU12, and OKT10 are used to construct immunotoxins. HB22.7-saporin was cytotoxic against a panel of non-Hodgkin’s lymphoma (NHL) cell lines and was shown to significantly prevent tumor development in a xenograft model of NHL [21]. HB2-saporin, BU12-saporin, and OKT10-saporin were shown to be selectively cytotoxic toward human acute lymphoblastic leukemia in vitro and in vivo [22–24].

Luster et al. [25] have reported that treatment with rGel-BLyS, rGel fused to a B-lymphocyte stimulator, rapidly reduced the tumor burden and markedly prolonged survival in xenograft mouse models of spread lymphoma or leukemia; in this setting, cell death was not induced by caspase activation but rather was partially mediated by the ribotoxic stress response. Furthermore, the rGel-BLyS fusion toxin combined with the proteasome inhibitor bortezomib restrained lymphoma growth and down-regulated nuclear factor kappa B (NF-κB) activity, which is critical for cellular proliferation and survival [26].

### Effects of RIPs on hepatoma and other cancers

MAP30 was shown to display anti-tumor activity in cell cultures and mice. In HepG2 cells, for example, cell viability was inhibited by MAP30 in time- and dose-dependent manners, with S-phase arrest; moreover, apoptosis and necrosis induced by MAP30 resulted in tumor volume reduction in HepG2-bearing mice [27]. Cucurmosin induced G_0_/G_1_ arrest and apoptosis in HepG2 cells; these effects also translated into potent anti-tumor activities in vivo [28]. Abrus agglutinin not only activates the caspase cascade but also suppresses Akt phosphorylation and NF-κB expression in HepG2 cells [29]. The effects of RIPs on other cancers are summarized in Table 1.

**Table 1:** Anti-tumor activities of various ribosome-inactivating proteins (RIPs)

RIP	Tumor type	Tested cell line(s)
Type I		
Trichosanthin	Breast cancer	MDA-MB-231^a^ and MCF-7 [5]
	Lymphoma	CEM, Hut-78, Raji, and Daudi [12]
	Cervical cancer	HeLa [37]; Caski [38]
	Choriocarcinoma	JAR and BeWo [39]
	Colon cancer	CT-26 [40]; LoVo [41]
	Hepatoma	HepG2 [42]
	Leukemia	Molt-4 and Jurkat [12]; K562 [43]
	Lung cancer	3LL^a^ [44]
	Melanoma	B16 [45]
	Nasopharyngeal cancer	CNE1^a^ and CNE2^a^ [46]; CNE2 [47]
	Prostate cancer	RM-1 [48]
	Gastric cancer	MCG803 [49]
α-Momorcharin	Breast cancer	MCF-7, EMT-6^a^, and MDA-MB-231^a^ [11]
	Colon cancer	SW480 and SW620 [50]
	Epidermoid	A431 and Hep-2 [50]
	Hepatoma	Hep G2 and SMMC-7721 [50]
	Lung cancer	NCI-H460 and A549 [50]
	Melanoma	B16, M14, SK-MEL-28, and A2058 [50]
	Nasopharyngeal cancer	CNE2 and HONE1 [51]
Momordica anti-HIV protein of 30 kDa	Bladder cancer	5637 [52]
	Breast cancer	MDA-MB-231^a^ [6]; BT20 [53]; MCF-7 [54]
	Epidermoid	A431 [53]
	Glioma	U87MG [53]
	Hepatoma	Hep G2^a^ [27]; Hep-3B [53]
	Melanoma	Malme-3M [53]
	Myeloma	U266 [53]
	Neuroblastoma	SK-N-SH [53]
	Prostate cancer	DU145 [53]
	Lung cancer	A549 [55]
Cucurmosin	Lung cancer	A549 [13]
	Melanoma	B16 [13]
	Hepatoma	HepG2^a^ [28]
	Leukemia	NB4 [14]; K562^a^ [56]
	Myeloma	RPM18226 [57]
	Pancreatic cancer	BxPC-3 [58]; SW-1990 [59]; PANC-1^a^ [60]; CFPAC-1 [61]
Saporin	Leukemia	NALM-6^a^ [22]; HSB-2^a^ [23]; CCRF CEM^a^ [24]
	Glioma	U87MG [62]
	Lymphoma	Ramos, Raji^a^, Daudi, DOHH-2, and Granta 519, SUDHL-4 [21]; HDLM2, KM/H2, and L428 [63]
	Neuroblastoma	SK-N-MC^a^ [64]
	Ovarian cancer	PA-1^a^ [64]
	Melanoma	SK-Mel-1^a^ [64], SK-Mel-28 [65]
	Pancreatic cancer	BxPC-3^a^ [66]
	Prostate cancer	LNCaP^a^, CWR22Rv1, and DU145 [67]; PC-3^a^ [68]
Gelonin	Breast cancer	MDA-MB-231^a^, BT-474, SKBR3, MCF-7, and Eb1 [8]
	Melanoma	MDA-MB-435^a^, WM35, WM46, WM3211, WM1346, WM1361A, WM1366, WM793, WM983A, WM983B, MeWo, SB2, A375, A375M, SK-MEL-1, SK-MEL-3, SK-MEL-5, SK-MEL-24, SK-MEL-28, SK-MEL-32, WM35P2N1, AAB-527, and Sbcl2 [18]
	Cervical cancer	ME-180 [69]
	Ovarian cancer	SKOV3 [69]
	Pancreatic cancer	Capan-1, Capan-2, MIA-PaCa-2, AsPC-1, BxPC-3, and L3.6P1 [69]
	Sarcoma	HT-1080 [69]
	Gastric cancer	NCI N-87 [69]
	Bladder cancer	T-24^a^ [69]; RT112^a^ [70]
	Epidermoid	A431 [71]
	Glioma	U87 MG [69]; 9L [72]
	Prostate cancer	PC-3 [72]
	Colon cancer	HT-29^a^ [71]; CT26^a^ and LS174T [72]
	Leukemia	NALM-6^a^ [25]; HL-60 [73]
	Lung cancer	Calu-3 [69]; A549^a^, H1975, and HCC827 [74]
	Lymphoma	Rec-1^a^ and NUDHL-1^a^ [25]; Mino^a^, JeKo-1, SP53 [26]; OCI-Ly3, OCI-Ly10^a^, SUDHL-4, and SUDHL-6 [75]
Marmorin	Breast cancer	MCF-7^a^ and MDA-MB-231^a^ [10]
α-Sarcin	Astrocytoma	251-MG [76]
	Breast cancer	MCF-7 [76]
	Glioma	RuGli [76]
	Pancreatic cancer	Patu II [76]
	Bladder cancer	EJ [77]
	Colon cancer	HT29 and BCS-TC2 [76]; SW1222 [78]
	Sarcoma	HT-1080 [76]; S-180 [79]; RD [80]
Curcin	Lung cancer	NCL-H446 [81]
	Gastric cancer	SGC-7901 [81]
	Sarcoma	S-180 [82]
α-Luffin	Breast cancer	MCF-7 [83]
	Choriocarcinoma	JEG-3 [83]
	Hepatoma	HepG2 [83]
MCP30	Prostate cancer	LNCaP, PC-3, and PIN [84]
Gelonium anti-HIV protein of 31 kDa	Breast cancer	MDA-MB-231^a^ [6]
Type II		
Riproximin	Breast cancer	MCF-7 and MDA-MB-231 [62]
	Larynx cancer	Hep2 [62]
	Leukemia	AR230, CML-T1, HL-60, LAMA84, SKW-3, K562, and BV173 [62]
	Lung cancer	NCI-H460 and Lewis^a^ [62]
	Pancreatic cancer	ASML^b^ [62]
	Prostate cancer	PC-3 [62]
	Sarcoma	Saos-2 [62]
	Cervical cancer	KB-3-1^a^ [62]; HeLa [85]
	Colon cancer	HT-29, CC531^b^, and CT-26^a^ [62]; HCT116 [86]
Abrus agglutinin	Hepatoma	HepG2^a^ [29]
*Momordica charantia* lectin	Nasopharyngeal cancer	CNE1 and CNE2 [35]
Articulatin-D	Leukemia	Jurkat, Molt-4, and HL-60 [15]
	Lymphoma	U937 and Raji [15]
Mistletoe lectin I	Leukemia	NALM-6 [87]
Foetidissimin II	Cervical cancer	HeLa [88]
	Leukemia	TF-1a [88]
Ebulin I & Nigrin b	Cervical cancer	HeLa [89]

^a^Cell lines that have been studied in mouse.

^b^Cell lines that have been studied in rat.

## Cellular mechanism of RIPs

### Entry mechanism

Ribosome-inactivating proteins must enter cells to inactivate the eukaryotic ribosome via their RNA *N*-glycosidase activity. First, type II RIPs bind to glycoproteins and/or glycolipids on the cell membrane and enter the cell via endocytosis; then, RIPs undergo retrograde transport from the Golgi apparatus to the endoplasmic reticulum via an intracellular pathway [30]. The enzymatic moieties will not be released to cytosol and reach the ribosomes to exert their function until they exploit the endoplasmic reticulum-associated degradation pathway [3].

**Figure 1:**
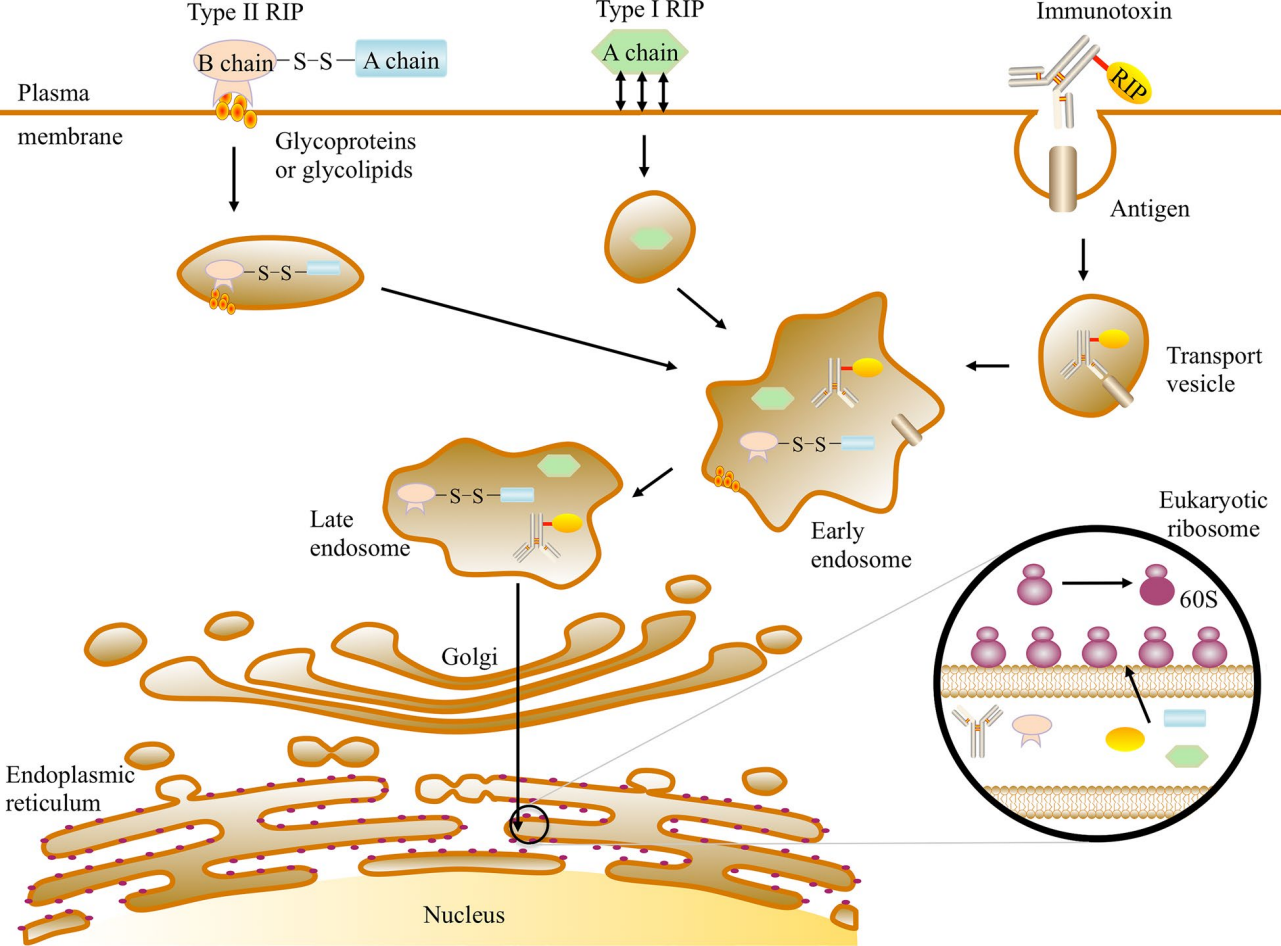
Cell entry mechanism of ribosome-inactivating proteins (RIPs). Different types of RIPs enter the cell through endocytosis and are subsequently degraded in the endoplasmic reticulum. They inactivate ribosomes through cleavage of the A_4324_ *N*-glycosidic bond, resulting in protein synthesis blockade.

It is difficult for type I RIPs to enter cells because of their sugar-binding activity deficiency. They can enter cells to some extent, probably due to their interaction with phospholipids in the cell membrane; however, the exact entry mechanism remains unclear. To facilitate the entry of type I RIPs into cells, they can be linked to proper carriers such as monoclonal antibodies and other molecules. The resulting conjugates can be specifically toxic to target cells. Several immunotoxins have been well studied in experiment therapies against hematologic and solid tumors. The entry pathways of type I RIPs, type II RIPs, and immunotoxins are shown in Figure 1.

### Induction of apoptosis in tumor cells

**Figure 2:**
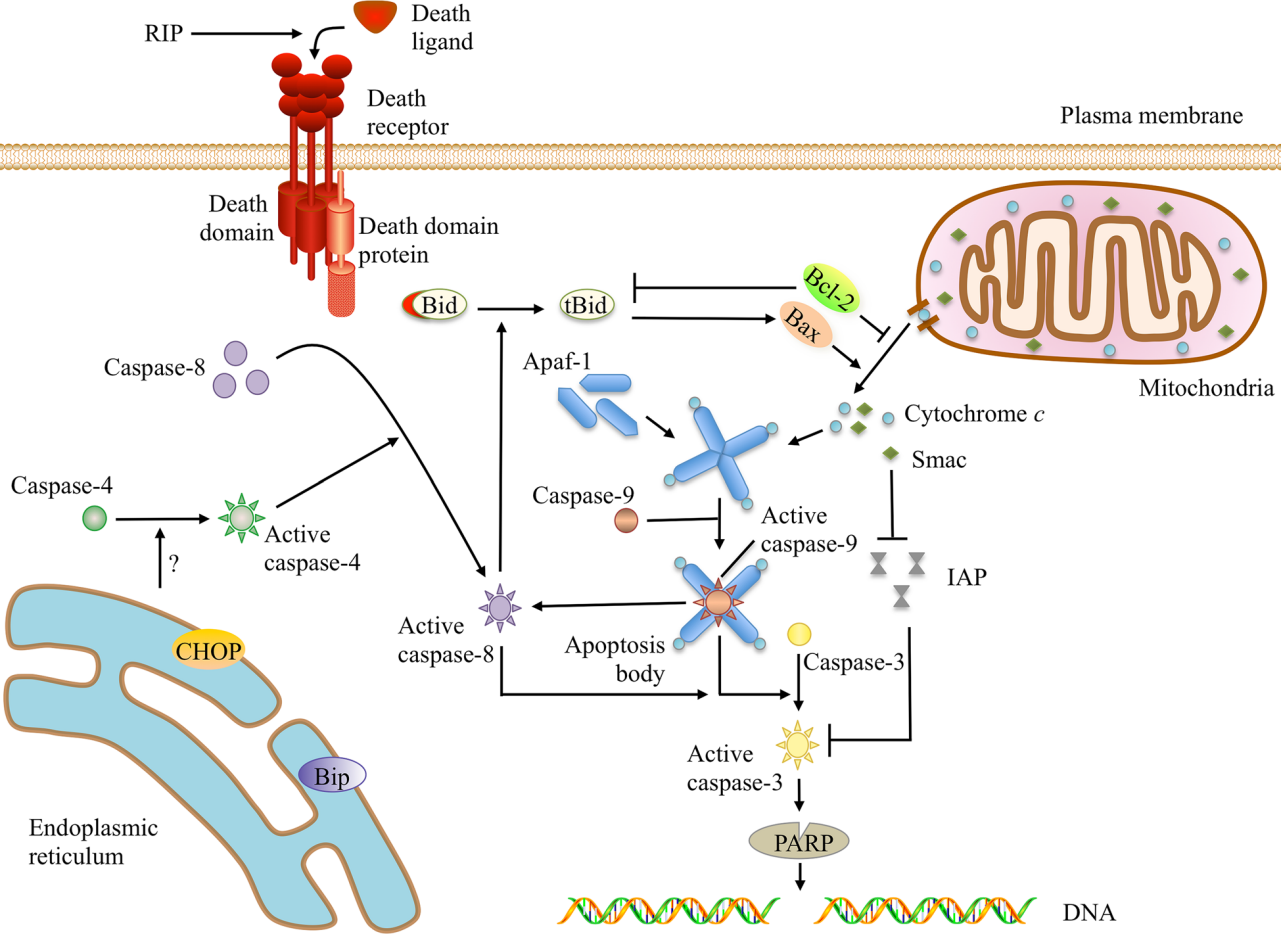
The apoptotic mechanism of RIPs. RIPs may trigger the death receptor pathway by facilitating the combination of the death ligand and its receptor. Caspase-8 is recruited and activated by death domain proteins such as Fas-associated protein with death domain (FADD). C/EBP homologous protein (CHOP) and immunoglobulin-binding protein (Bip) are increased under RIP-induced endoplasmic reticulum stress, in which activated caspase-4 contributes to capase-8 activation. The release of second mitochondria-derived activator of caspases (Smac) and cytochrome *c*, which can be increased by Bax or decreased by Bcl-2, is promoted by RIP. Cytochrome *c* aggregates with apoptotic protease-activating factor 1 (Apaf-1) and becomes an apoptotic body that activates caspase-9, which in turn activates caspase-3 and caspase-8. Activated caspase-3 cleaves poly(ADP-ribose) polymerase (PARP), resulting in DNA fragmentation and apoptosis. Smac protects caspase-3 from inhibitor of apoptosis protein (IAP) inhibition. Caspase-8 cuts Bid into tBid, which is necessary for Bax oligomerization in the mitochondrial outer membrane. The inhibition of tBid insertion into the mitochondrial membrane by Bcl-2 prevents cytochrome *c* release [31].

Caspases play an important role in apoptosis. They are classified into three types: initiator, executioner, and cytokine processor caspases. Great progress has been made in studying the three signaling pathways related to caspase activation, including mitochondrial, death receptor, and endoplasmic reticulum stress signaling pathways. The connections among these pathways are shown in Figure 2 [31]. Apoptosis also occurs through apoptosis-inducing factor (AIF), which is caspase-independent [32].

#### Mitochondria-mediated apoptosis

Recent studies have indicated that apoptosis-inducing substances can lead to excessive reactive oxygen species production, intracellular Ca^2+^ imbalance, and a series of pathologic changes, resulting in mitochondrial membrane potential and permeability changes. Then, the pro-apoptotic factors cytochrome *c*, AIF, second mitochondria-derived activator of caspases (Smac), and apoptotic protease-activating factor 1 (Apaf-1) are released from the mitochondria to participate in the process of apoptosis.

Mitochondrial membrane potential depolarization and caspase-9 activation were detected in MCF-7 cells and to a lesser extent in MDA-MB-231 cells after marmorin treatment [10]. Li et al. [33] reported the loss of mitochondrial membrane potential (the point of no return in apoptotic cascades) in HL-60 cells after apoptosis was induced by TCS. In addition, Orrenius et al. [34] noted that cytochrome *c* release is dominated by the Bcl-2 family of proteins. Furthermore, simultaneous Bax up-regulation, Bcl-2 down-regulation, and poly(ADP-ribose) polymerase (PARP) cleavage were noted in Abrus agglutinin-treated HepG2 cells, caspase-3/7 activity levels failed to increase after Bax knockout, and Bcl-2-overexpressing hepatocellular carcinoma cells were found to be ricin-resistant [29].

Several pumps, such as the Na^+^–K^+^ pump and the Ca^2+^ pump, maintain concentration gradients of various ions to achieve appropriate membrane potential. Alterations in the mitochondrial membrane potential after the induction of apoptosis lead to changes in membrane permeability. The results mentioned above suggest that changes in mitochondrial membrane permeability could cause apoptosis, which is induced by RIPs through decreasing the Bcl-2/Bax ratio (modifying the outer mitochondrial membrane permeability); this in turn enhances cytochrome *c* and Smac translocation into the cytoplasm and activates caspase-9 and the downstream executioner caspase-3, thereby increasing the production of cleaved PARP and resulting in DNA fragmentation and apoptosis [34–36].

#### Death receptor-mediated apoptosis

Death receptors, such as Fas, deliver apoptotic signals into the cytoplasm by binding to Fas ligand (FasL); the signals are then passed to downstream procaspase-8, the activation of which demands the cytoplasmic adaptor molecule, which is indispensable to the binding and proteolysis of procaspase-8 for activation. Once activated, the initiator caspase-8 can activate caspase-3, eventually leading to cell apoptosis.

Marmorin was found to trigger the death receptor apoptotic pathway in MCF7 cells; this pathway is also preferentially activated in MDA-MB-231 cells [10]. Due to caspase-3 deficiency in MCF7 cells, caspase-8 amplifies the apoptotic signal through cleavage of the protein Bid, which punctures the mitochondria and causes mitochondrial collapse, thereby generating sufficient effector caspase levels. Conversely, TCS does not affect Fas or FasL levels, indicating that the Fas/FasL pathway is not involved in TCS-induced apoptosis [32].

#### Endoplasmic reticulum stress-mediated apoptosis

Endoplasmic reticulum stress is found in cells exposed to environmental toxins, hypoxia, viruses, ultraviolet light, and other stimuli. Its manifestations include misfolded and/or unfolded protein aggregation in the endoplasmic reticulum lumen as well as Ca^2+^ balance disorders. Endoplasmic reticulum stress can promote a series of physiologic changes in the endoplasmic reticulum. Accumulated misfolded and/or unfolded proteins are processed, allowing cells to maintain their normal functions and remain alive. However, excessive endoplasmic reticulum stress can cause apoptosis.

Trichosanthin treatment was shown to up-regulate the endoplasmic reticulum stress-related proteins Bip (immunoglobulin-binding protein) and CHOP (C/EBP homologous protein) in HL-60 cells, thereby activating caspase-4, which is involved in caspase-3 activation [89]. Endoplasmic reticulum stress was also described in marmorin-treated MCF7 and MDA-MB-231 cells, as evidenced by CHOP up-regulation and caspase-12 cleavage [10].

Horrix et al. [86] identified activation of the unfolded protein response (UPR) in response to endoplasmic reticulum stress; the UPR is induced in MDA-MB-231 cells exposed to low concentrations of the type II RIP riproximin. As many cancer cells activate the UPR to cope with stressors, α-MMC was shown to down-regulate the UPR in NPC cells; however, substantial apoptosis was not observed until the α-MMC dosage reached a certain threshold, indicating that α-MMC at low concentrations probably inhibit increased cell generation via down-regulation of the UPR [51]. There are two conceivable strategies to initiate apoptosis through endoplasmic reticulum stress: (1) prolonging the UPR to induce apoptosis, which likely occurs in riproximin-induced apoptosis; and (2) blocking the UPR so that tumors are vulnerable to stressors, as with α-MMC.

## Future research emphasis

Conventional cancer drugs that are currently in use often lack tumor specificity, which greatly limits the therapeutic dose and curative effect. A feasible way to overcome this issue is the use of targeted therapy, as follows: (1) suitable targeted delivery such as with the immunotoxins mentioned above or with bi-specific antibodies (containing two different specific antigen recognition Fab fragments); (2) a tumor-specific expression strategy, in which the cDNA of RIP is synthesized and cloned into a plasmid vector controlled by a cancer-specific promoter, eventually producing RIP in the cell cytoplasm. These strategies must be investigated in a series of preclinical studies before assays can be conducted in human subjects. Several saporin-containing immunotoxins in clinical trials have exhibited promising results [20], whereas other RIP-containing immunotoxins have barely been studied. A few factors must be considered when translating preclinical data into the clinic: the risk of immunogenicity and toxicity in patients should be minimized; the minimum effect dose and maximum tolerated dose should be determined; and possible adverse effects during treatment should be predicted. Tumor-specific expression strategies are rarely reported; therefore, this idea remains to be explored.

## Conclusions

Abundant evidence indicates that RIPs exert their cell-killing abilities through a variety of mechanisms, many of which are caspase-dependent. Although several mechanisms involved in RIP-induced apoptosis have been elucidated, more studies are required to reveal the precise mechanism. Considering the potential use of RIPs in important diseases and their effectiveness as immunotoxins for targeted therapy, RIPs are worthy of further exploration.
